# Murder of Dr. Braman, U. S. A.

**Published:** 1868-09

**Authors:** 


### Murder of Dr. Braman, U. S. A.

The sad death of this promising physician and esteemed contributor to this journal was mentioned in a recent issue. The following particulars of it have been received:

One of the Doctor's friends, Lieut. Clark, had been robbed of his watch and a considerable sum of money. Suspicion fell upon Second Lieutenant William McGee, an officer of the same regiment. Lieut. Clark, who is a member of the Masonic Fraternity, as also was Dr. Braman, informed the Doctor of his loss, and the Doctor expressed his doubts of Lieut. McGee. Their suspicions coming to the knowledge of Gen. Sykes, the commanding officer of the garrison, he ordered an investigation, but nothing was proved against McGee. Dr. Braman then wrote a letter to Gen. Sykes, regretting his action; said that his zeal as a Mason led him to help a brother Mason (Lieut. Clark;) that in an interview with McGee, the latter removed his suspicions, and that he would do everything possible to counteract what had been done.—McGee, however, was not satisfied, and at dusk on the evening of the 15th ult., went to Dr. Braman's room with a revolver, and without any warning shot him while in the act of rising from his chair. Dr. Braman immediately ran to a hospital tent near by, and was placed on a bed, where he died. McGee is only 17 or 18 years of age. He entered the army as a drummer-boy, in the 33d New Jersey Regiment, and a year ago obtained a commission in the regular army. Men who have been discharged from his regiment represent him to be of dissipated habits, and often seen on duty under the influence of liquor. It is to be hoped that he will receive the punishment which so cowardly and brutal an action deserves.—*Medical and Surgical Reporter.*

